# Supplementary material for: Spatial Point Pattern Analysis Identifies Mechanisms Shaping the Skin Parasite Landscape in Leishmania donovani Infection
Source: Front Immunol. 2021 Dec 16;12:795554. doi: 10.3389/fimmu.2021.795554 (PMC8716623; doi:10.3389/fimmu.2021.795554)
Supplement: Supplementary file 1 [file DataSheet_1.pdf]

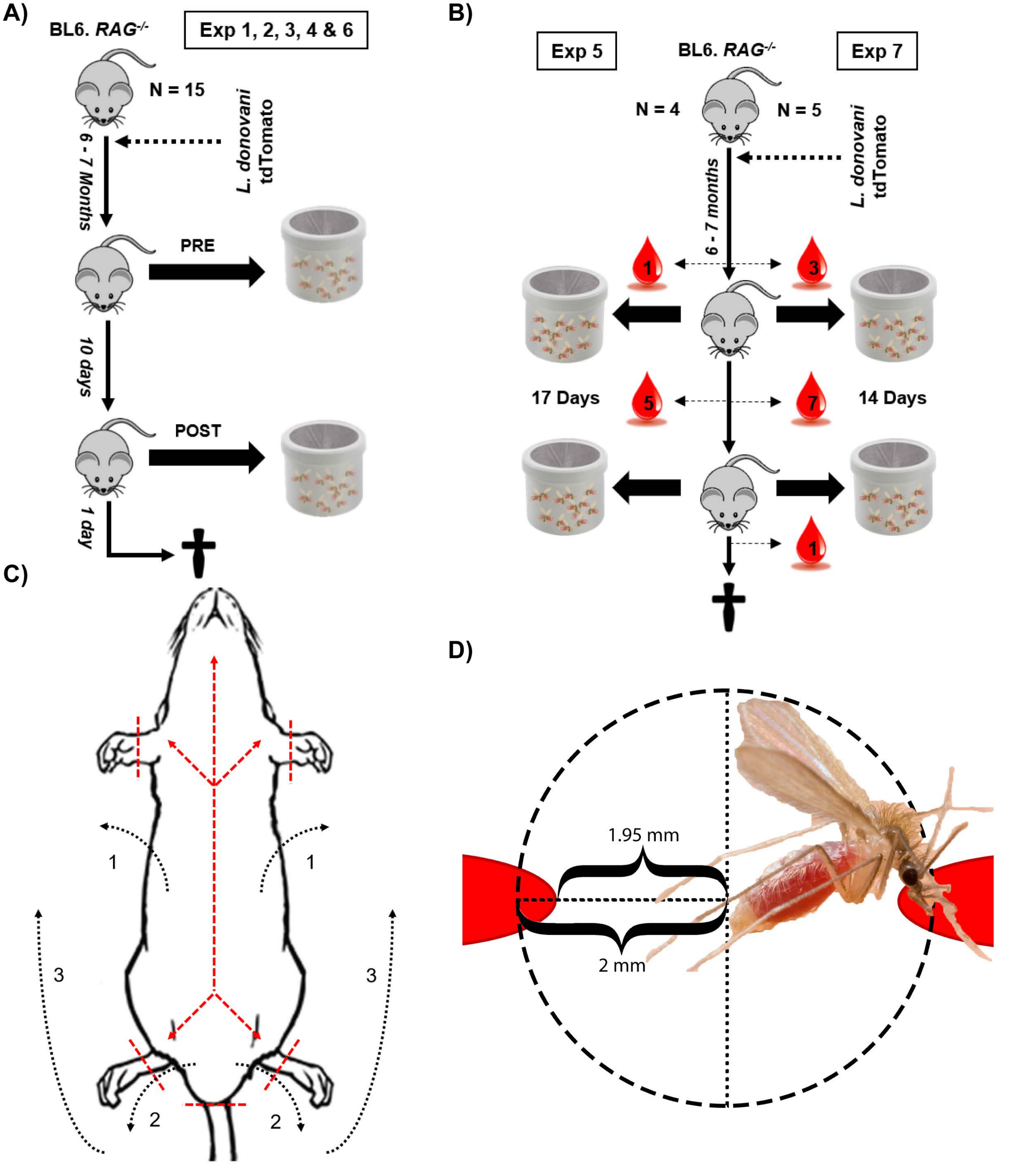

Figure S1 | Schematics of experimental designs and methodologies
(A) Experimental schematic representing the treatment of RAG1-10 & RAG15-18. (B) Experimental schematic representing the treatment of RAG11-14 (EXP5) & RAG19-23 (EXP7). (C) Schematic representation of technique for mouse skinning. (D) Schematic representation of the concept for the nearest-neighbor distance pattern analysis. If we pinned the sand fly down at its posterior and swung it once around, the proboscis tip would describe a circle, which would mark the most distal point the sand fly could bite irrespective of its orientation. Thus, any parasite patch reaching into this area could be potentially accessed by a sand fly without having to mave of its spot.

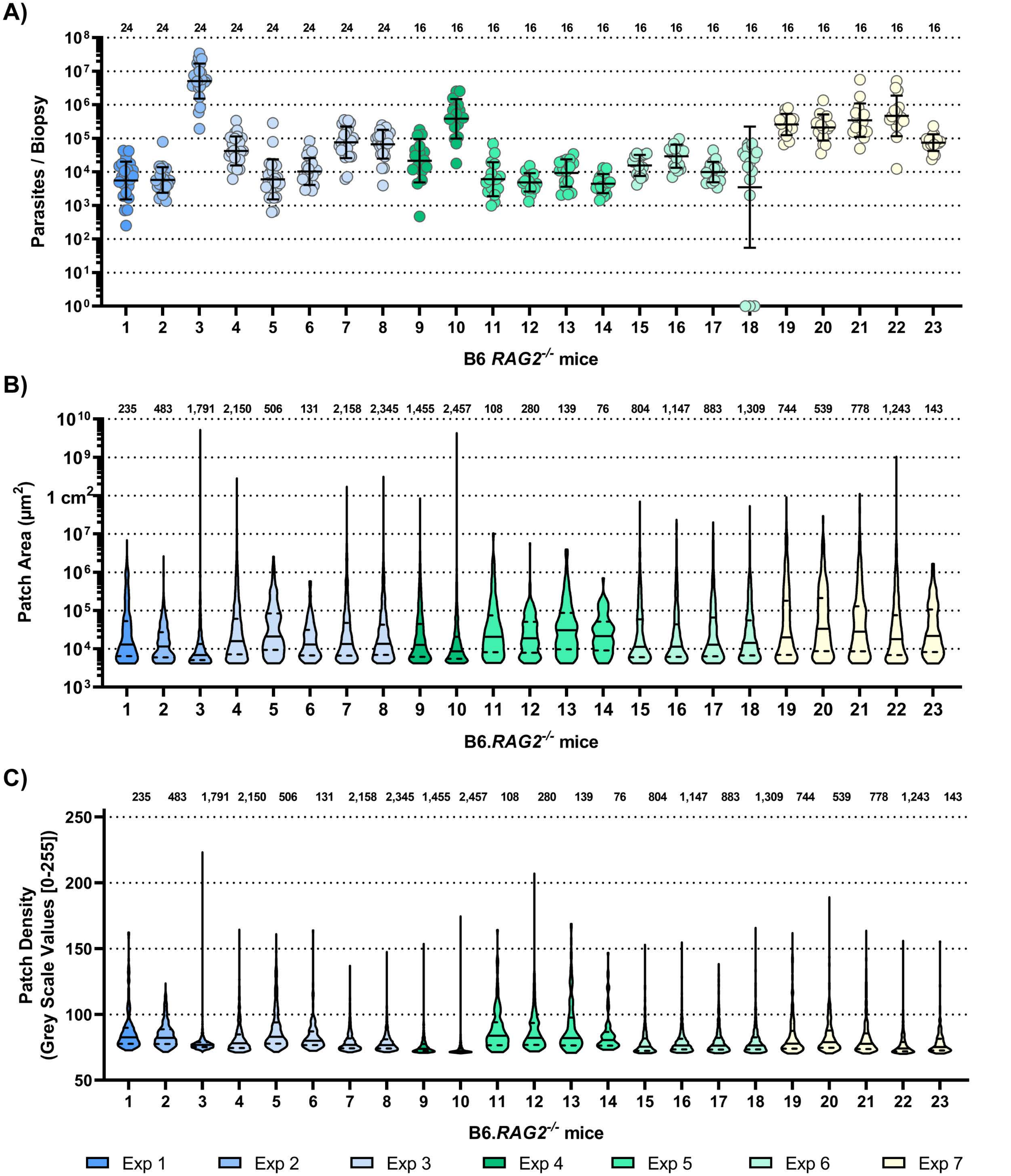

Figure S2 | Graphs from figure 1 resolved by individual RAG mouse

(A) Quantitative PCR data of estimated parasite loads of 24 or 16 biopsies (ø 0.6 cm) / RAG mouse. This graph shows the same data as in Fig. 1A resolved by individual RAG mice (Kruskal-Wallis Test: $P<0.0001$). (B) Skin parasite patch area data from Fig. 1B resolved by respective RAG mice (Kruskal-Wallis Test: $P<0.0001$). (C) Mean parasite patch density / patch as shown in Fig. 1F resolved by respective RAG mice (Kruskal-Wallis Test: $P<0.0001$).

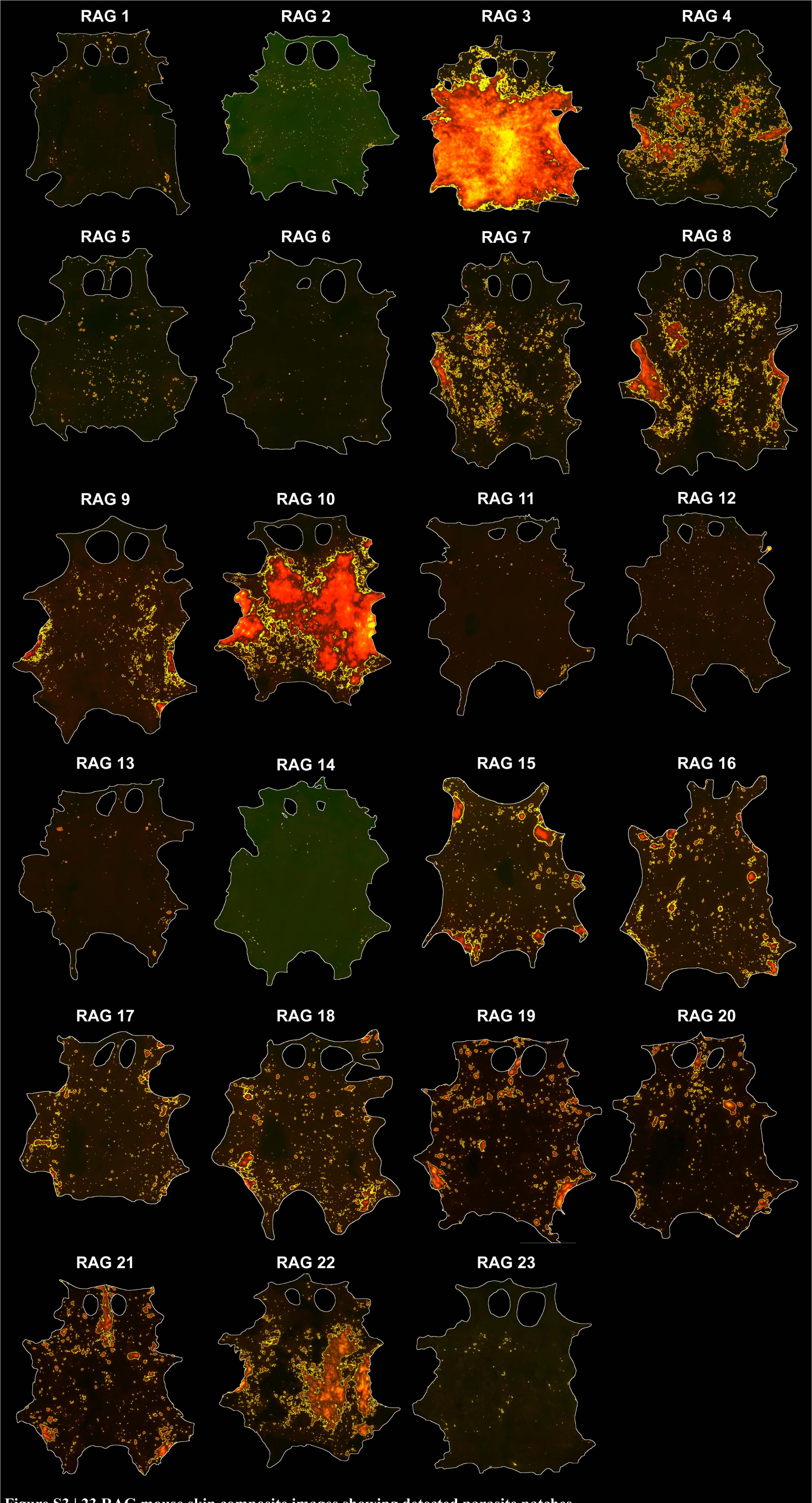

Figure S3 | 23 RAG mouse skin composite images showing detected parasite patches

Composite stereomicroscopic image of the L. donovani tdTomato-signal as described in the methods section. All detected skin parasite patches in the ImageJ analysis are framed by yellow outlines. Note: The Photoshop composite image for RAG2 and RAG14 caused an irreversible background error in the green channel when read into ImageJ, giving these two images a green hue in the output. However, all data acquisition was done in the red channel and thus, this error had no effect on the data collection.

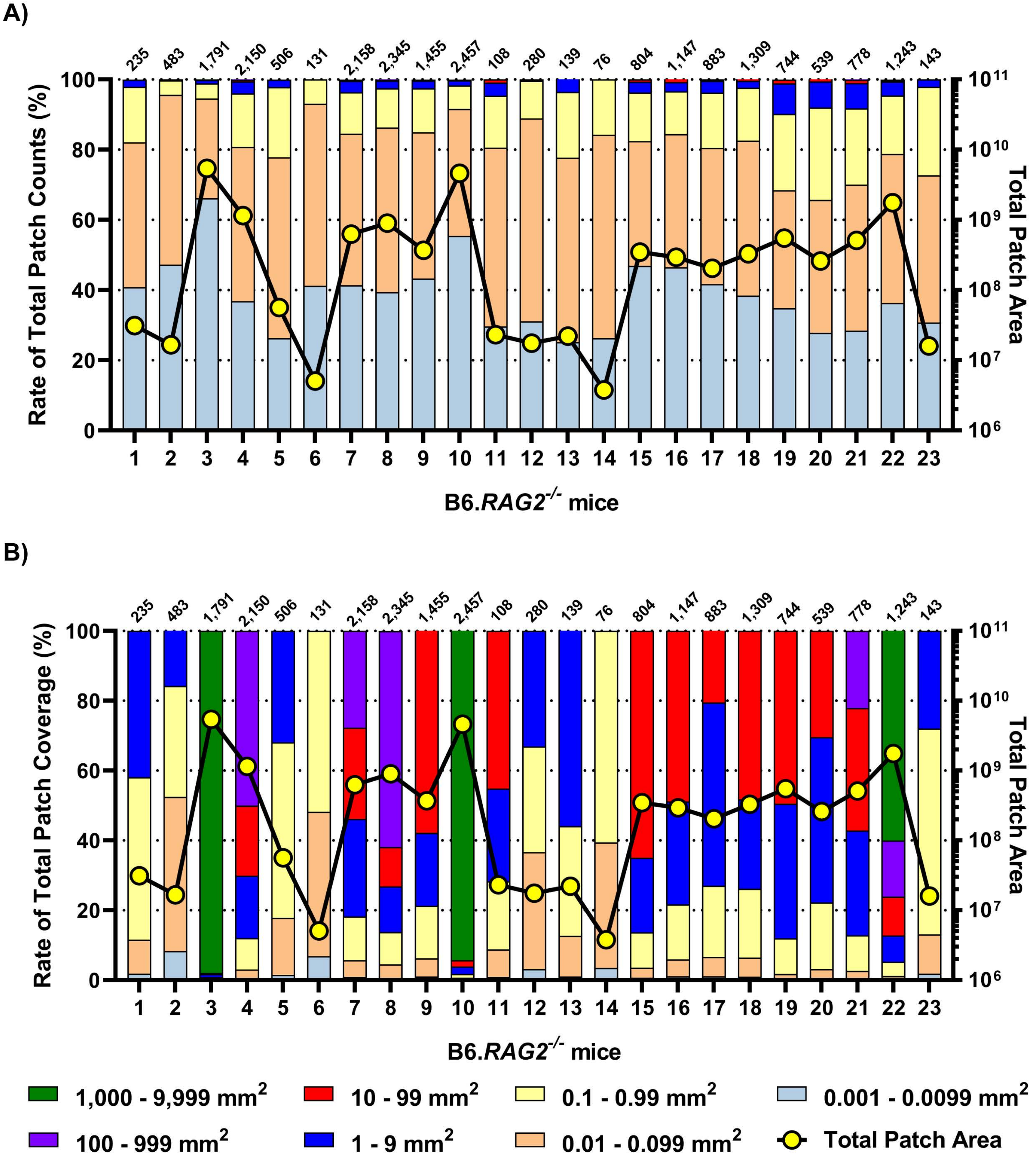

Figure S4 | Bar graphs from figure 1 E resolved by individual RAG mouse

(A) Bar chart of the ratio of patch counts / size category showing the same data as in Fig.1E 'A' resolved by the respective RAG mice (Friedman Test: P=0.7544). (B) Bar chart of the contribution of size category to the overall patch coverage showing the same data as in Fig.1E 'B' resolved by the respective RAG mice (Friedman Test: P=0.7345).

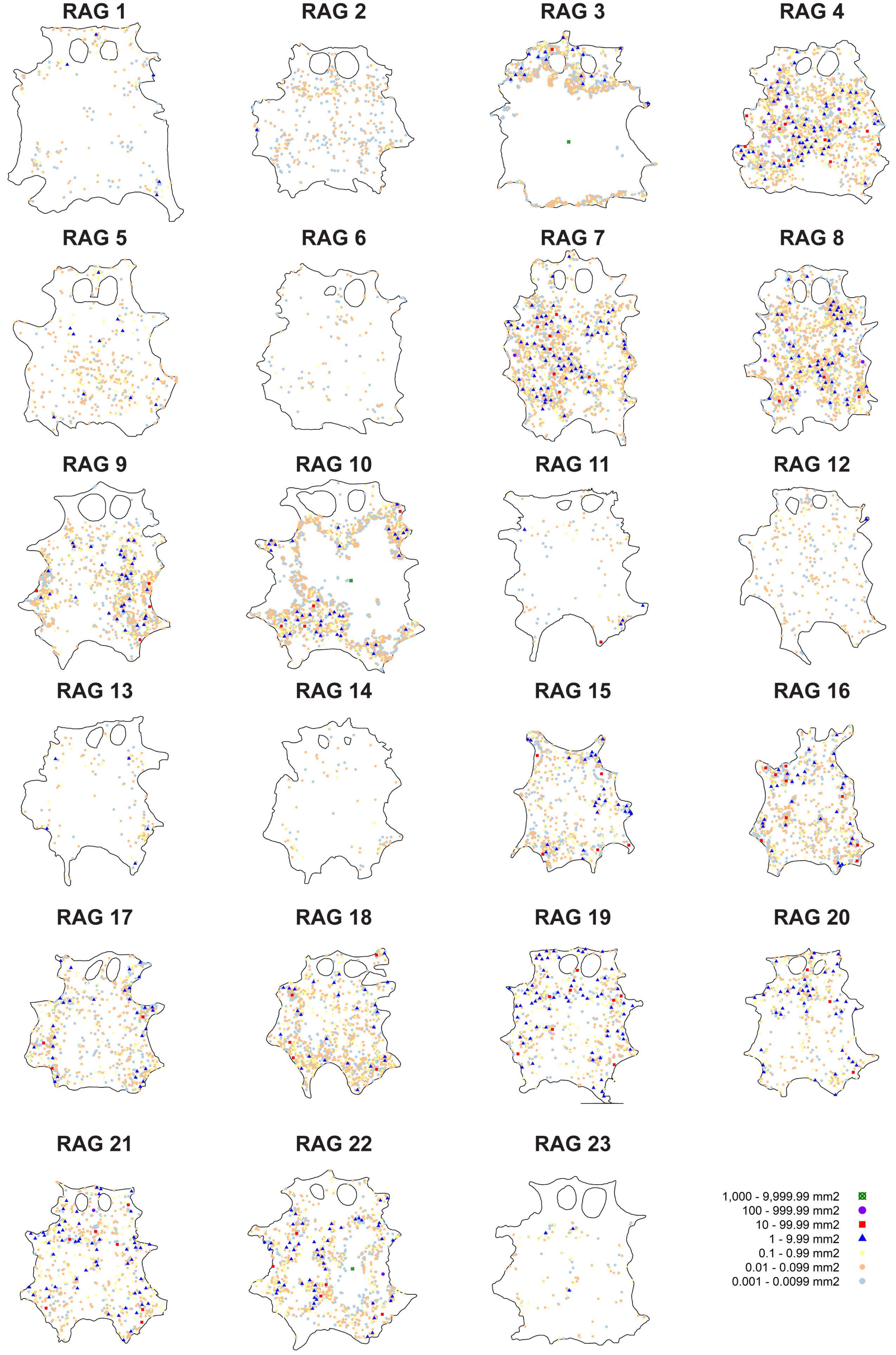

Figure S5 | Schematic distribution of skin parasite patches marked by patch area

Skin parasite patches were categorized by patch size according to the log-scale employed in Fig.1E and plotted according to the location of the center of mass for each skin parasite patch.

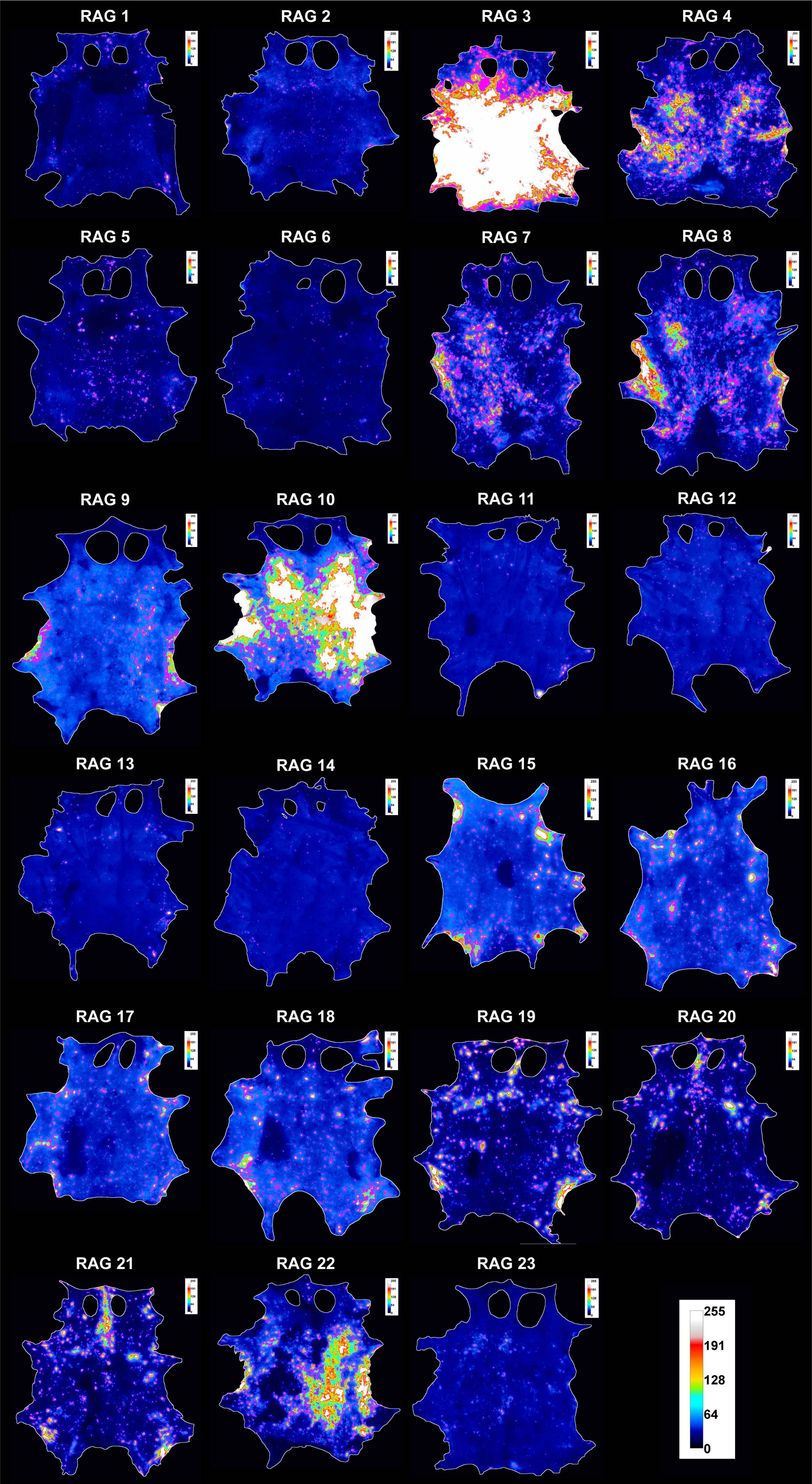

Figure S6 | Pixel density skin images

8-bit grey-scale images, like in Fig.1H, showing the tdTomato light intensity per pixel for each analyzed RAG mouse, respectively. Dark blue refers to lowest light intensity, while white refers to highest light intensity. See scale for reference. In magenta are the outlines of detected skin parasite patches.

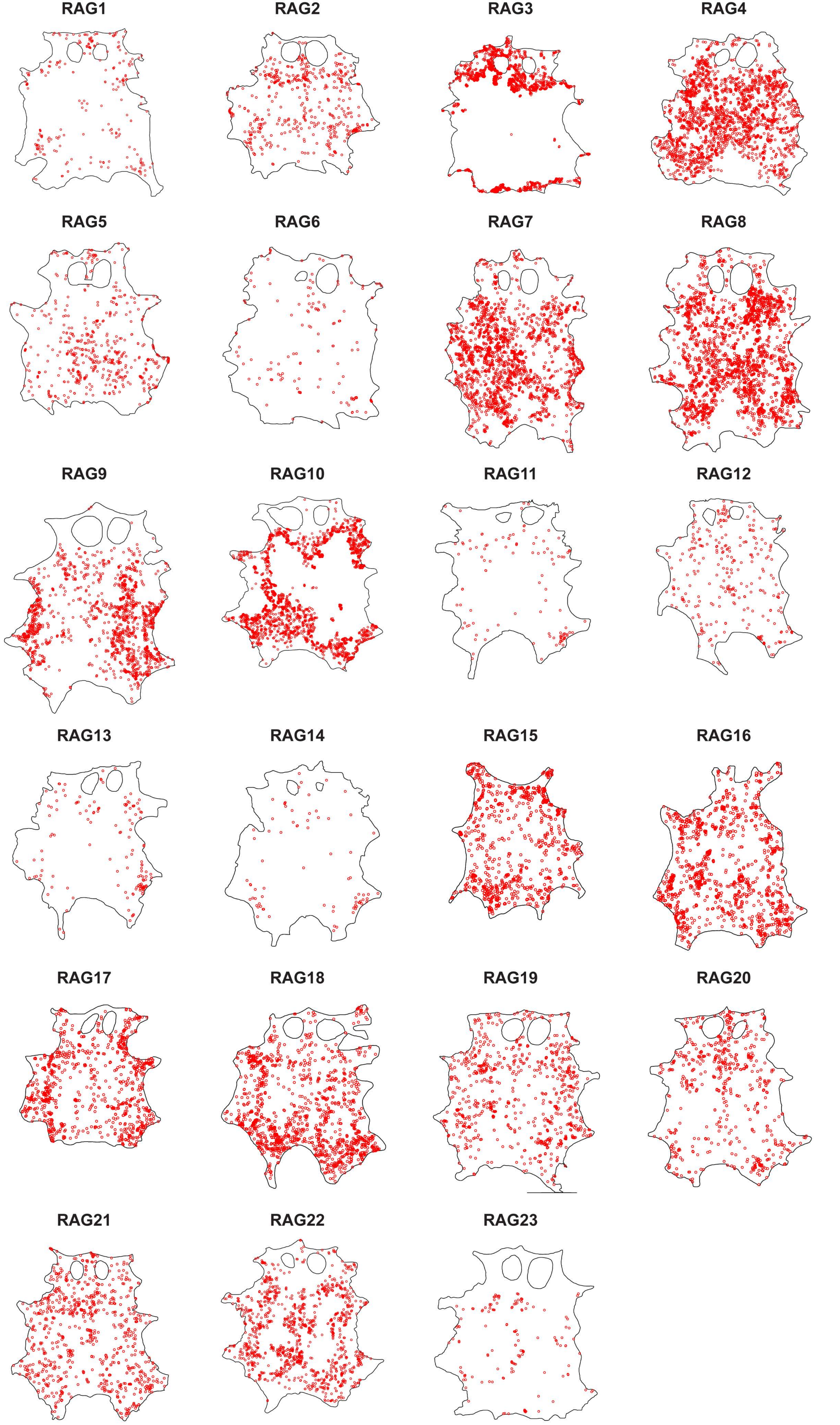

Figure S7 | Representation of skin parasite patches as spatial point pattern without marks
Each red circle marks the center of mass of parasite patches within the skin. The circle size does not convey any measure here.

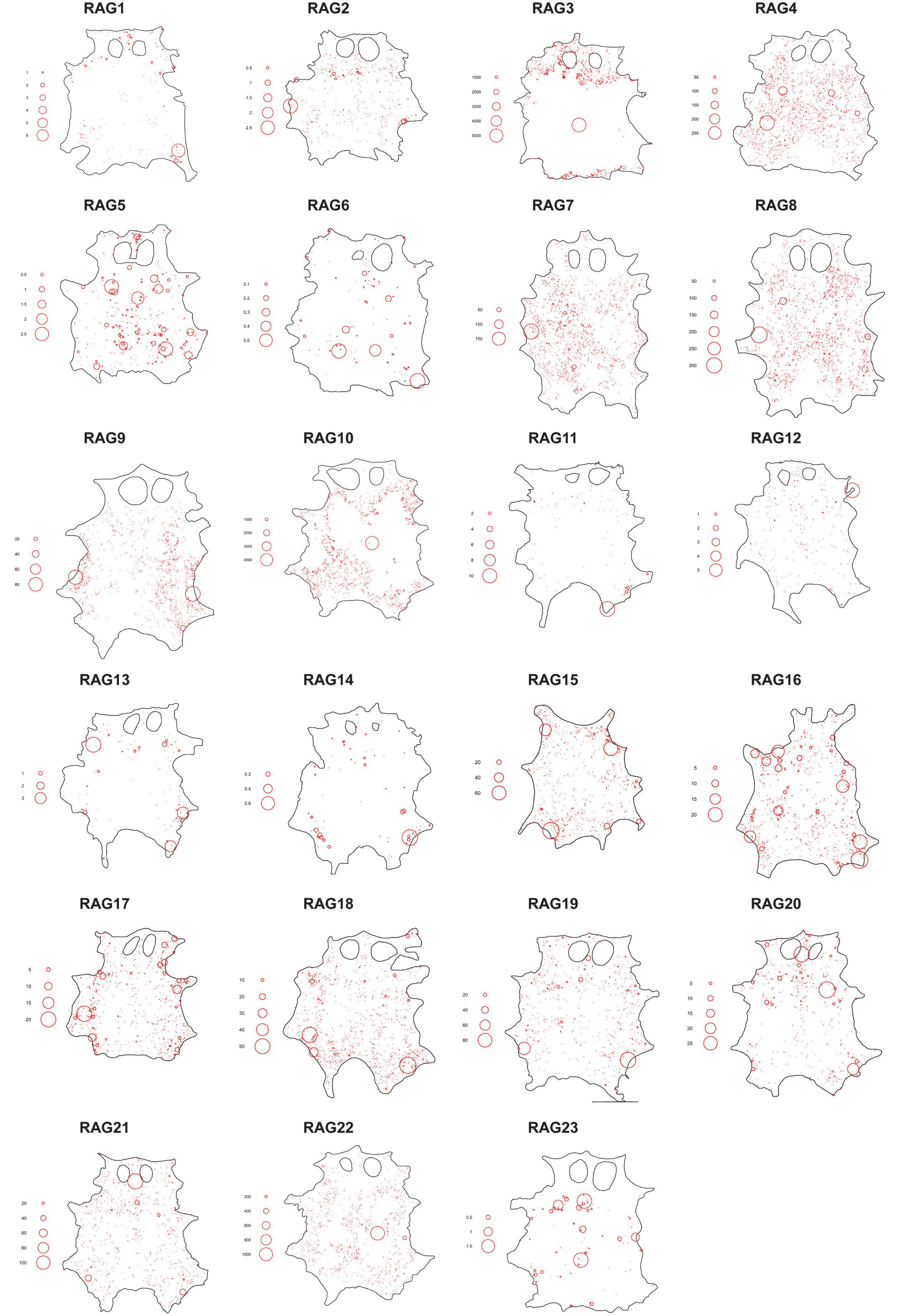

Figure S8 | Representation of skin parasite patches as spatial point pattern with area mark

Each red circle marks the center of mass of parasite patches within the skin. The circle size corresponds to the relative area of each patch without information on the shape of the patch. The circle size scale is specific to each graphical representation.

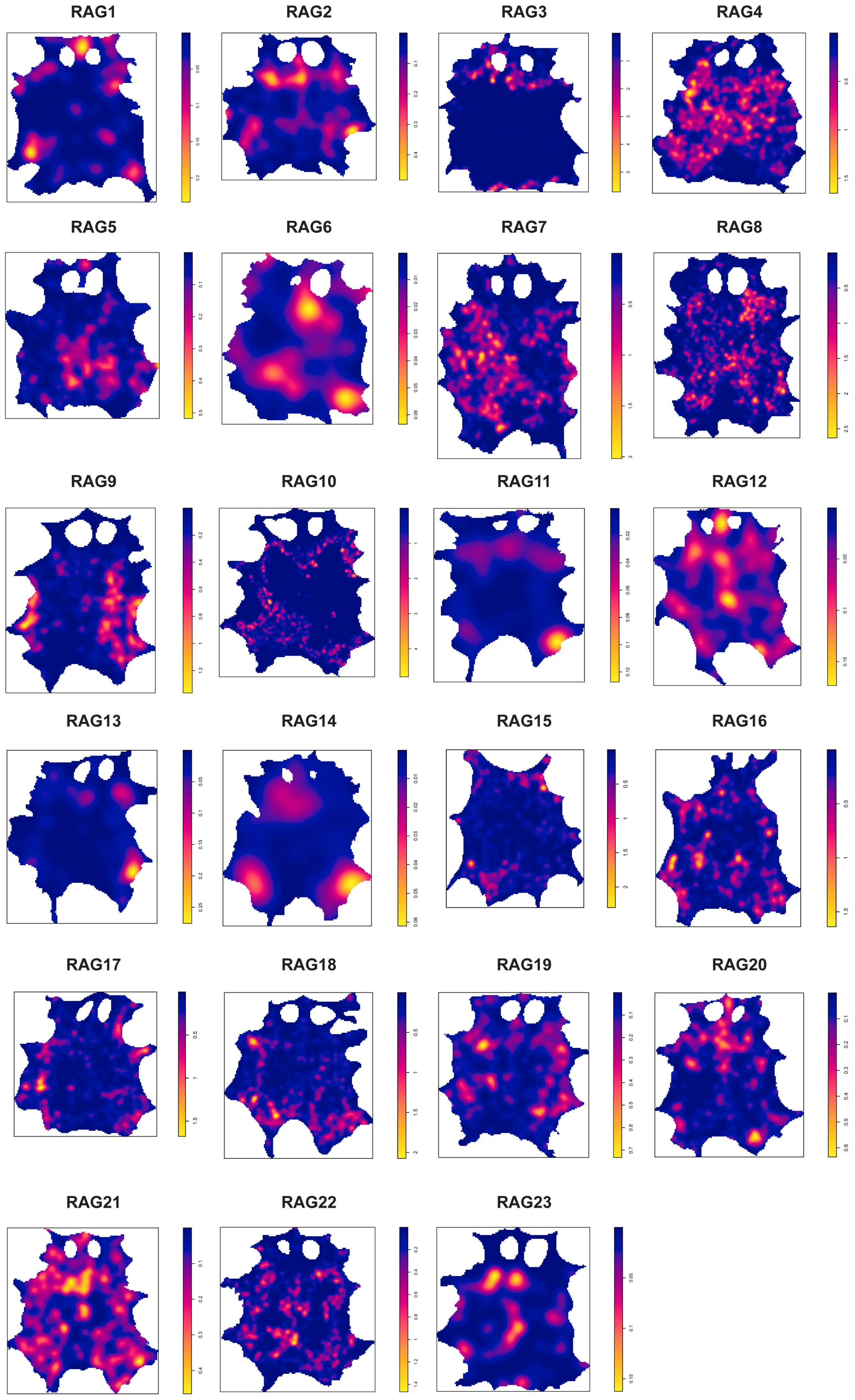

Figure S9 | Intensity measurement plots without marks

The images show how closely skin parasite patches are clustered together in different sites of the skin. These intensity measurements do not take account of patch size. On the color scale, blue-purple colors indicate low skin parasite patch density, while orange-yellow indicate high skin parasite patch density. The scale is relative to each image.

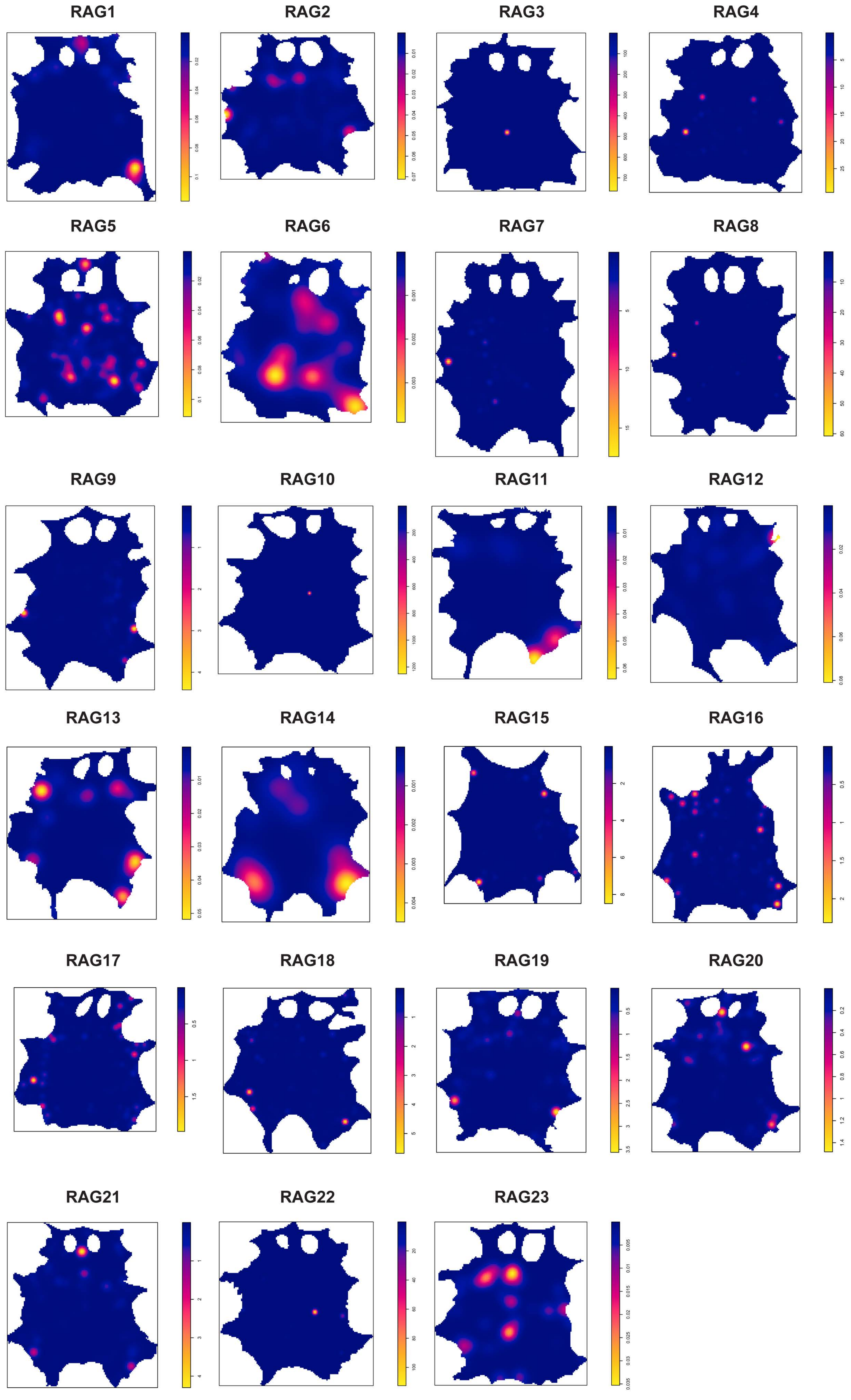

Figure S10 | Intensity measurement plots with area mark

The images show the distribution of skin parasite patches by patch area. On the color scale, blue-purple colors indicate the presence of skin parasite patch with small patch area, while orange-yellow indicate the presence of skin parasite patch with large patch area. The scale is relative to each image.

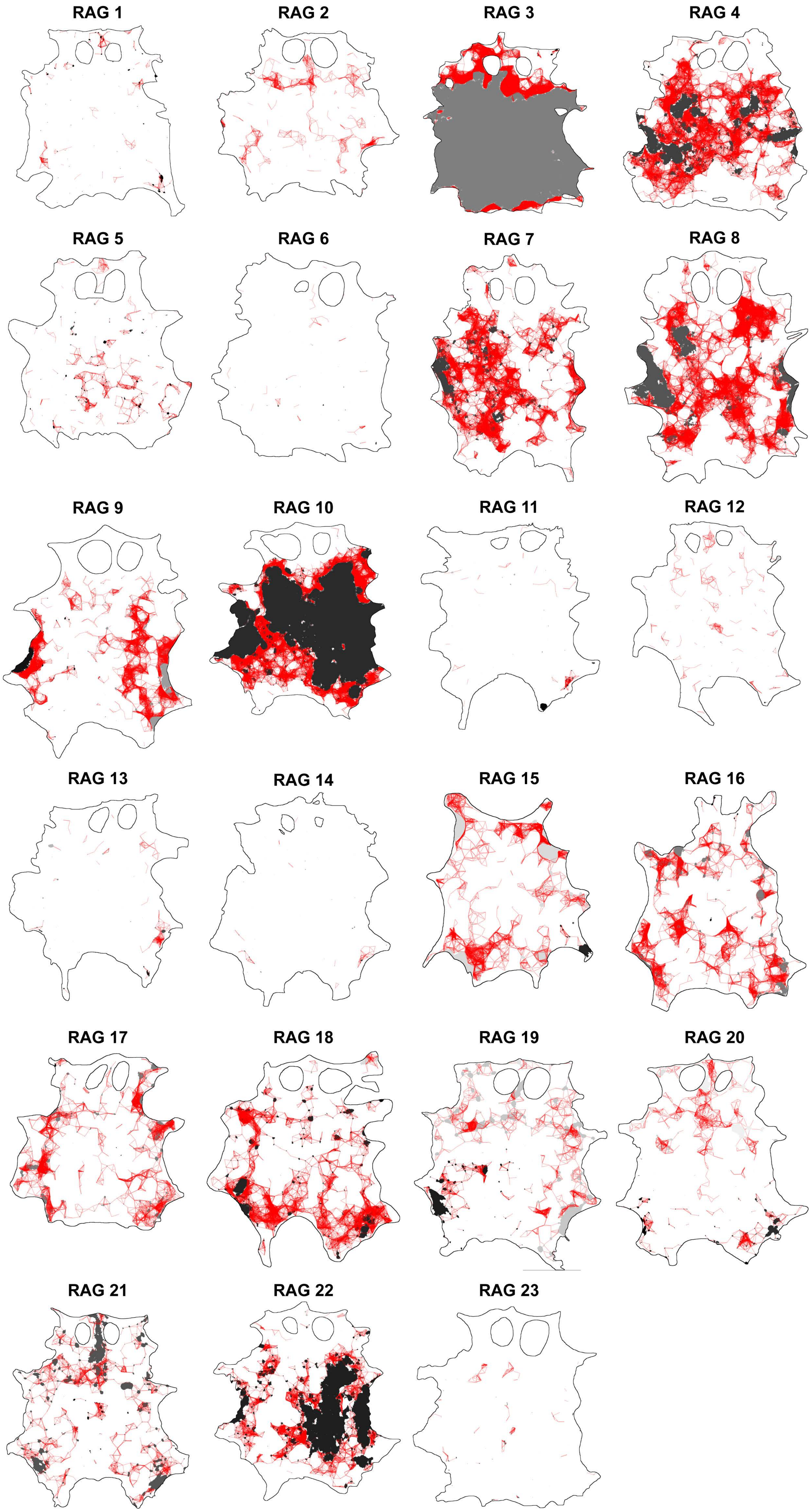

Figure S11 | Graphical representation of cluster analysis

The red lines indicate where patches are separated ≤3.9 mm from patch periphery to patch periphery. Individual patches are marked in different shadings of grey. The red lines indicate where networks of closely placed skin parasite patches may enhance the likelihood of biting sand fly finding a patch, while the shaded areas represent the patches.

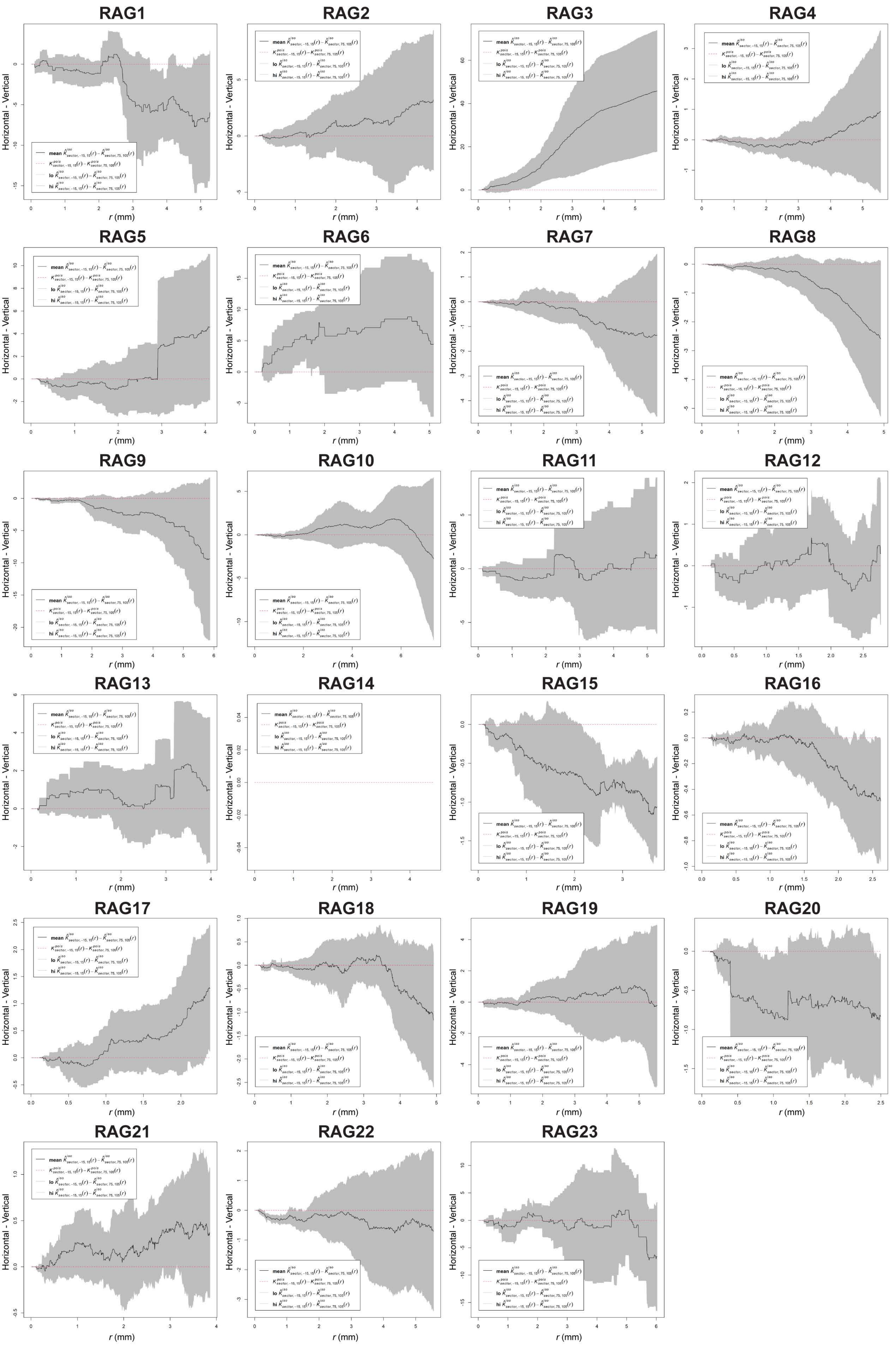

Figure S12 | Test for isotropy of skin parasite patch point patterns

The red dotted lines mark the theoretical homogeneous Poisson process as a null hypothesis. The black solid lines represent he plotted data by distance of patches in mm (r). The grey shaded area around the solid black line is the 95% confidence interval. Should the red dotted line be not included into the grey shaded area at any stage then the point pattern is not isotropic. RAG14 did not produce a result due to too few skin parasite patches for the analysis.

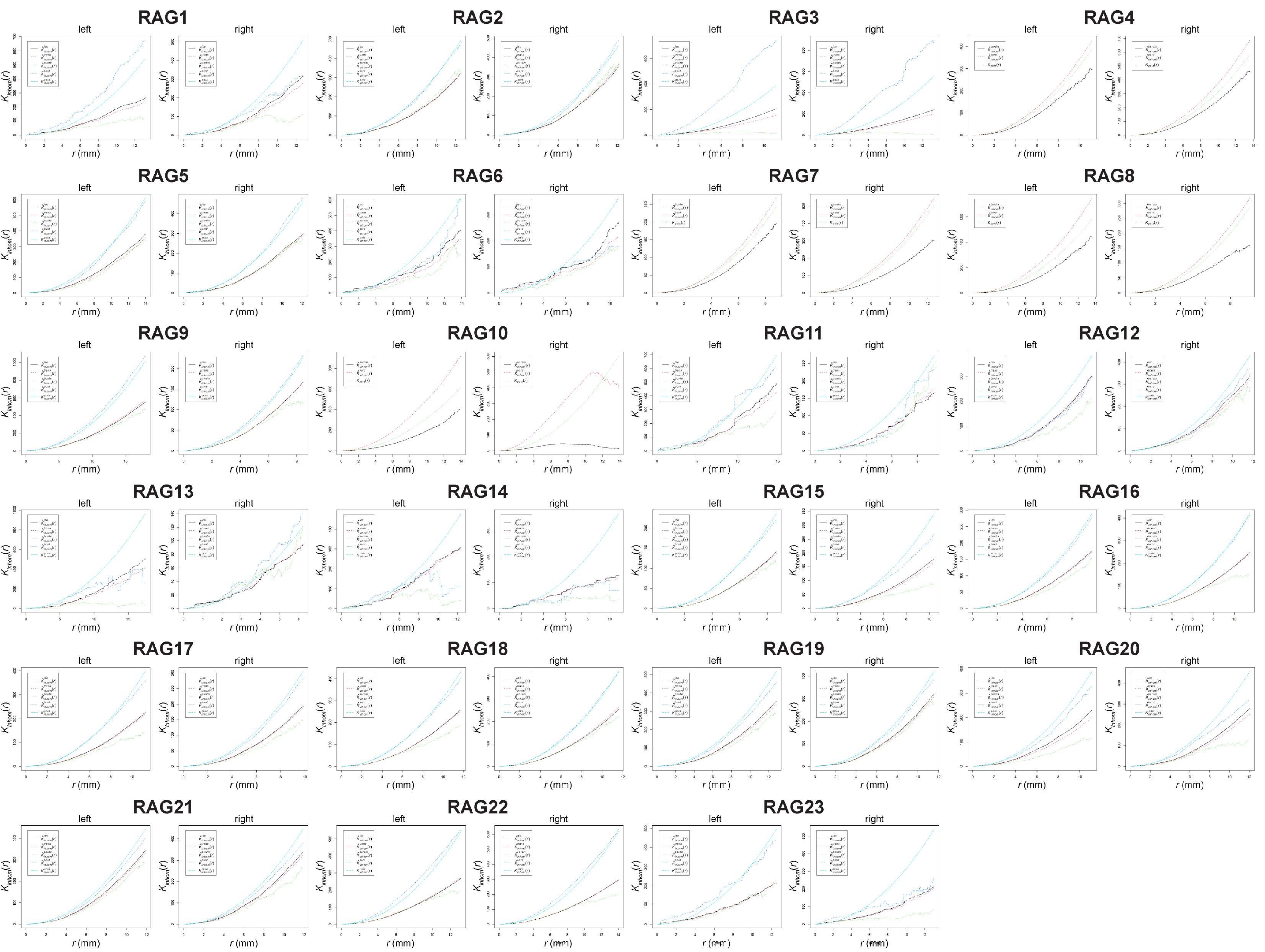

Figure S13 | Correlation-stationary test using the inhomogeneous K-function

The double graphs show the theoretical inhomogeneous Poisson process as a null hypothesis and up to four graphs based on different border correction approaches. Please, consult the respective graph legends for the color code of each graph.

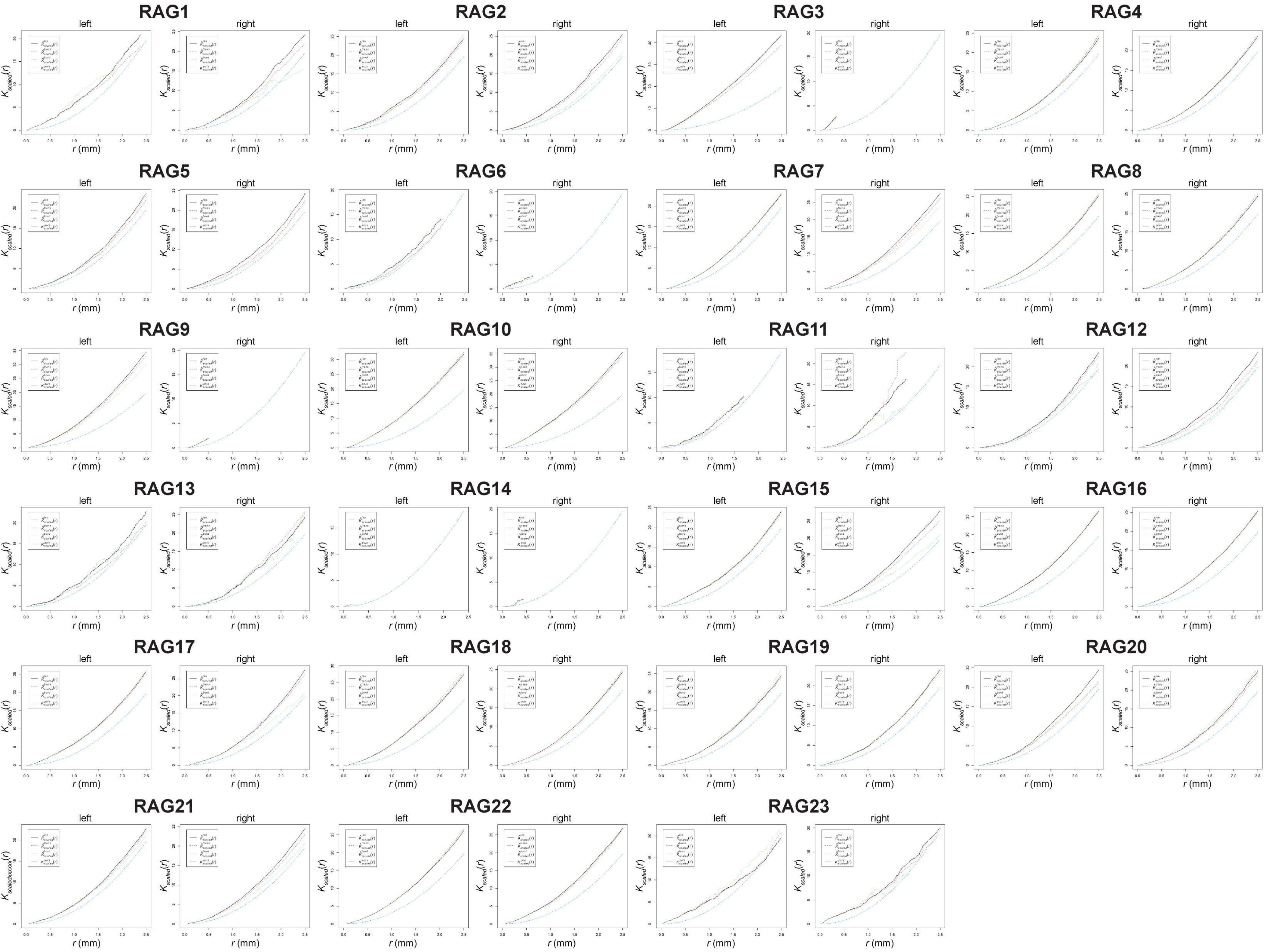

Figure S14 | Correlation-stationary test using the locally scaled K-function

The double graphs show the theoretical inhomogeneous Poisson process as a null hypothesis and up to four graphs based on different border correction approaches. Please, consult the respective graph legends for the color code of each graph.

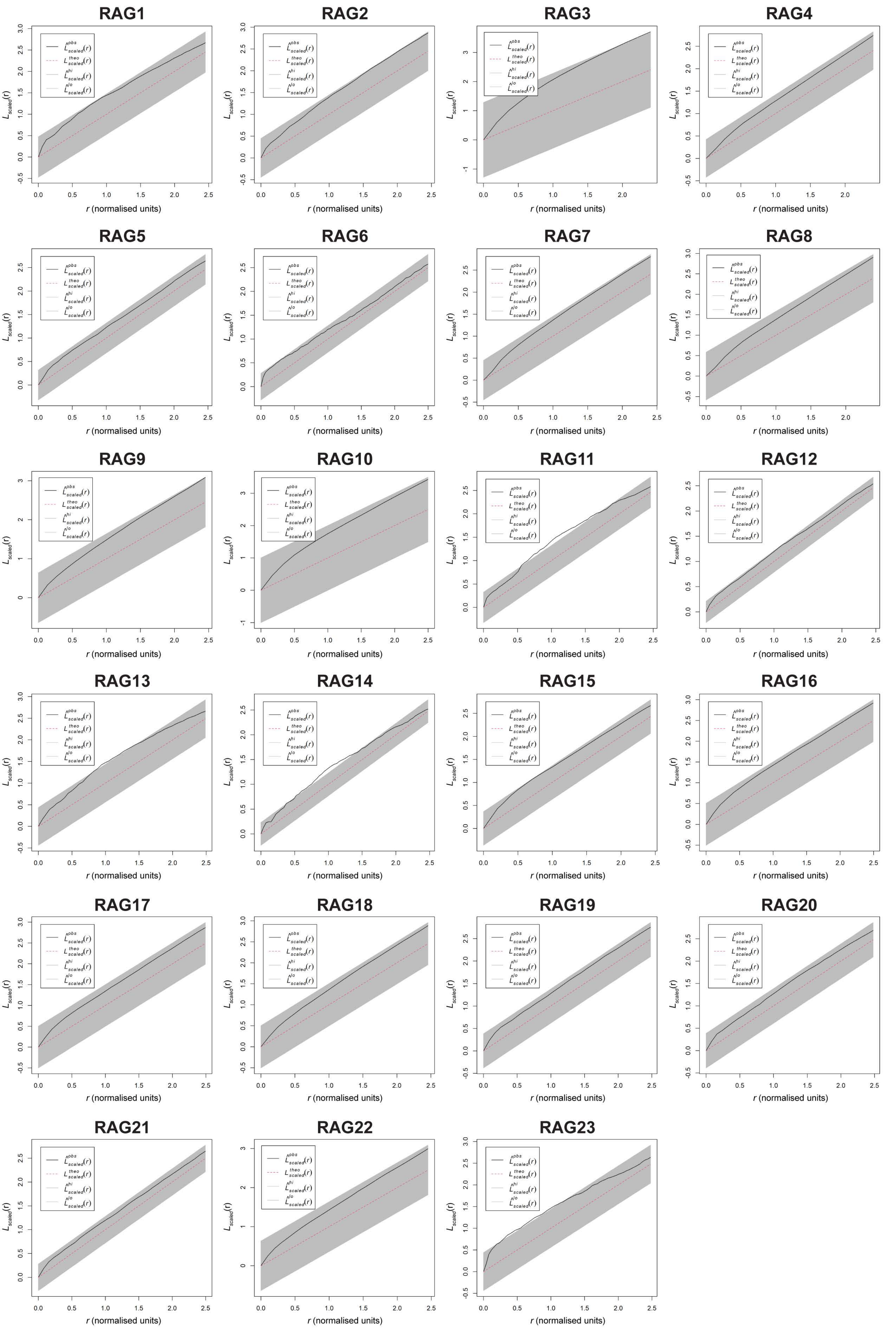

Figure S15 | Scaled L-function of skin parasite patch distribution

The red dotted line represents the theoretical locally scaled Poisson process as a null hypothesis. The solid black line shows the plotted data. The data is presented against the distance 'r' between skin parasite patches measured in mm. The grey areas are simulation-envelopes around the theoretical Poisson process. Departure of the solid black line above or below the red dotted line are indicative of clustered and regular distributions instead of an independent distribution, respectively. Departure of the solid black line from the grey shaded simulation-envelops indicates significant departure from the theoretical Poisson process.

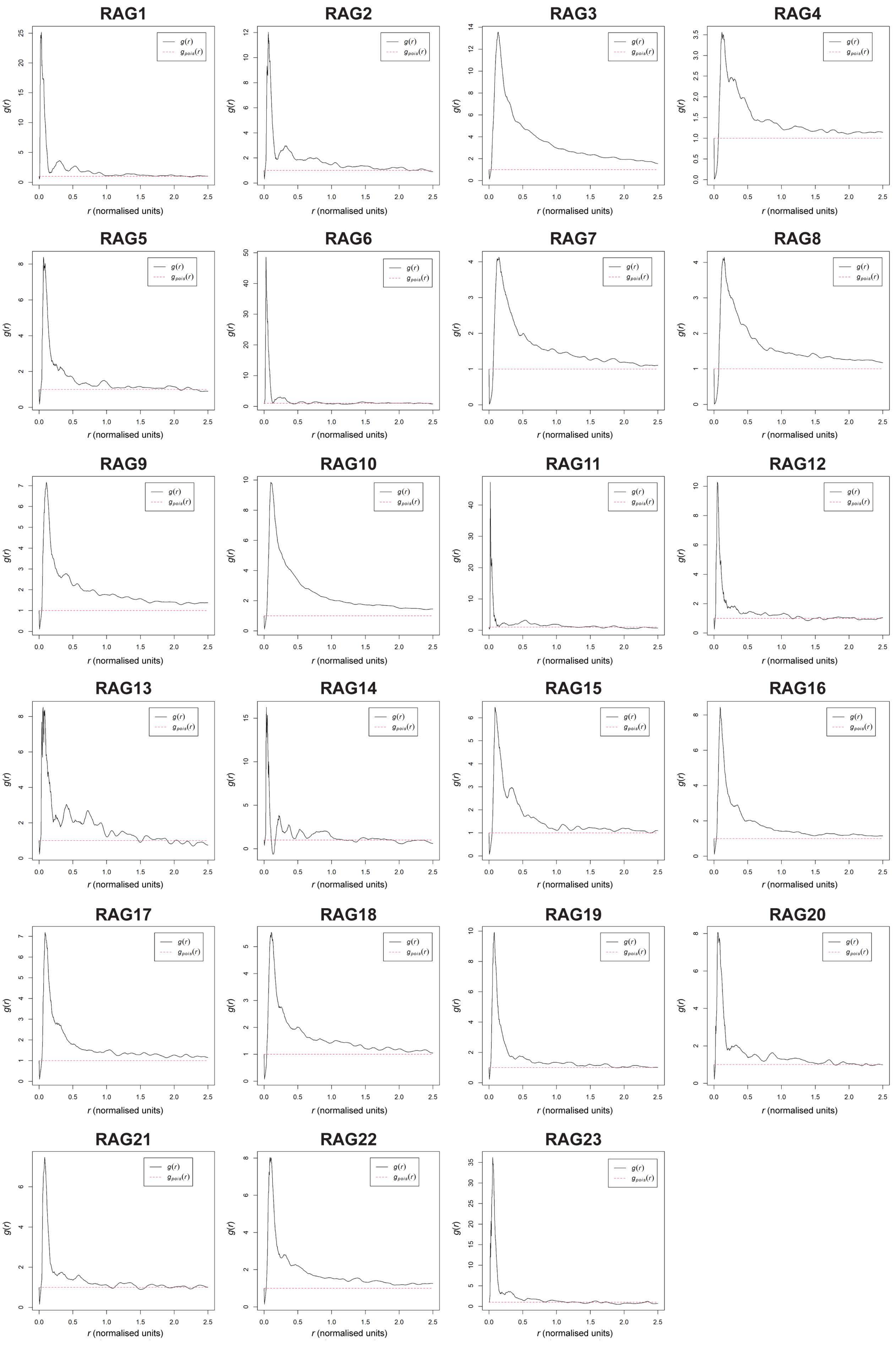

Figure S16 | Pair correlation function plots

The red dotted line represents the theoretical locally scaled Poisson process as a null hypothesis. The solid black line shows the plotted data. Departure of the solid black line above or below the red dotted line are indicative of clustered and regular distributions instead of an independent distribution, respectively.

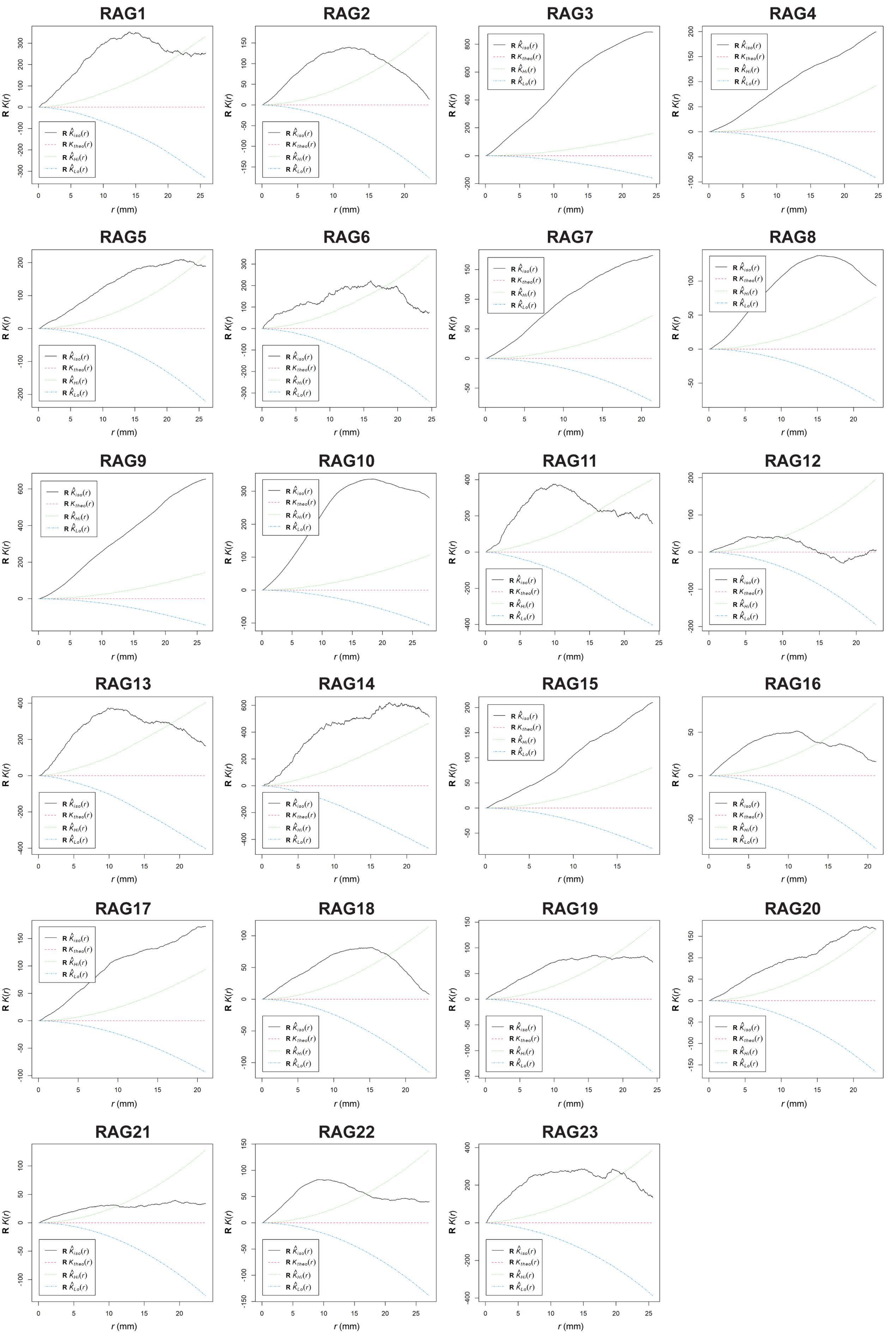

Figure S17 | K-function of point process residuals

The red dotted line represents the theoretical locally scaled Poisson process as a null hypothesis. The solid black line shows the plotted data. The blue and green line mark the limits of the upper and lower confidence interval. Departure of the solid black line above or below the red dotted line indicates significant departure of data point independence. Further, departure of the solid black line above or below the red dotted line are indicative of clustered and regular distributions instead of an independent distribution, respectively.

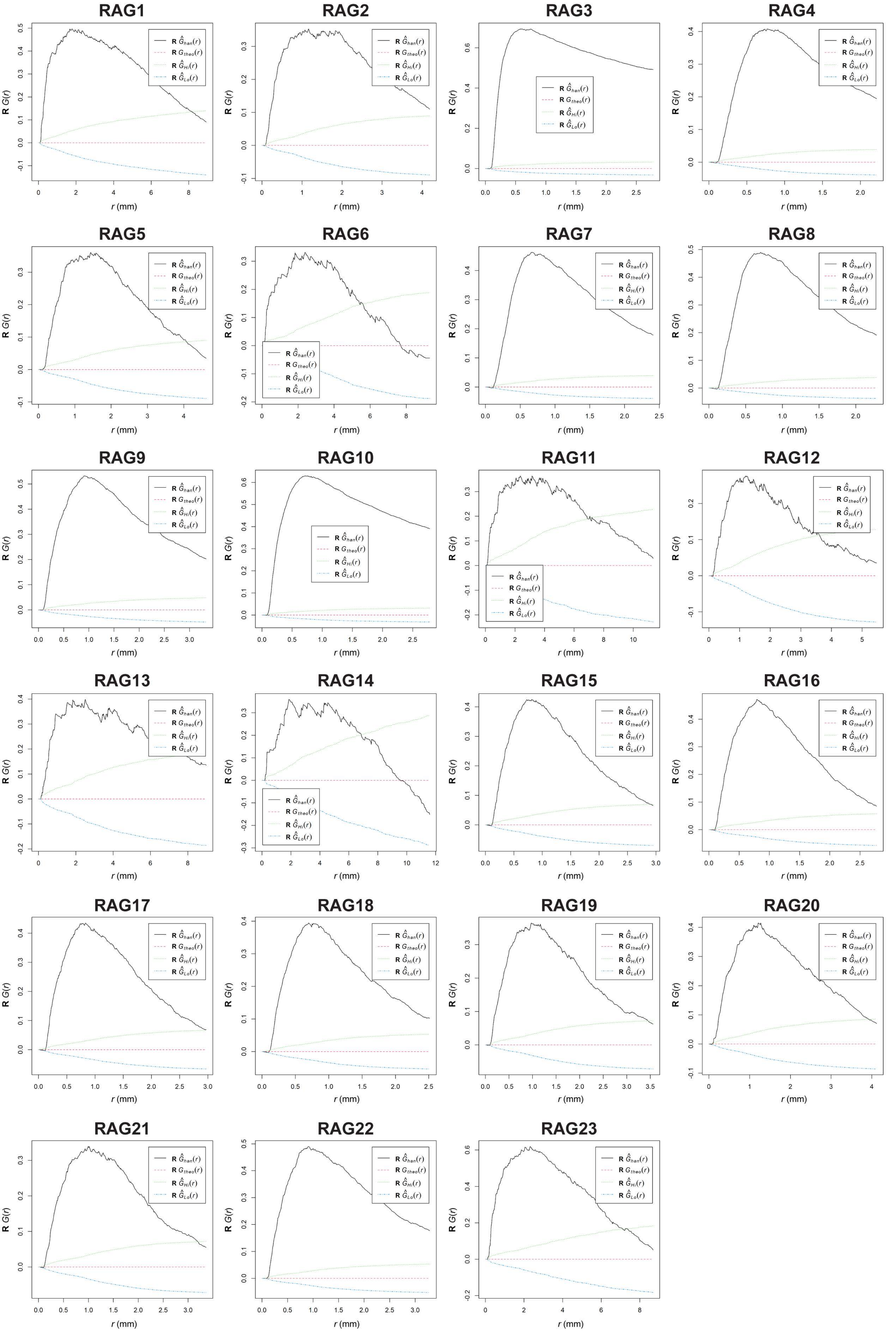

Figure S18 | G-function of point process residuals

The red dotted line represents the theoretical locally scaled Poisson process as a null hypothesis. The solid black line shows the plotted data. The blue and green line mark the limits of the upper and lower confidence interval. Departure of the solid black line above or below the red dotted line indicates significant departure of data point independence. Further, departure of the solid black line above or below the red dotted line are indicative of clustered and regular distributions instead of an independent distribution, respectively.

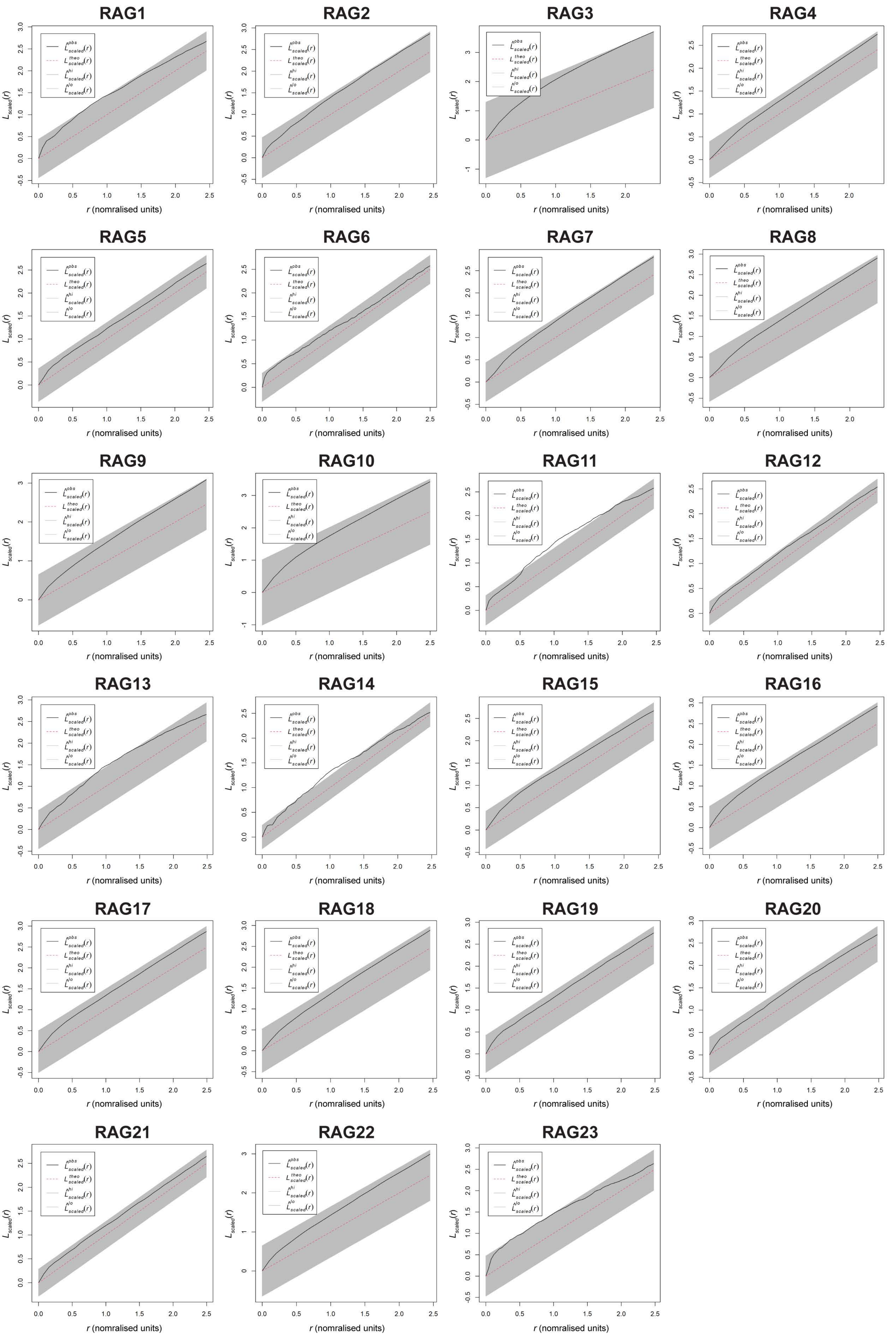

Figure S19 | Scaled L-function of simulated skin parasite patch distribution

The red dotted line represents the theoretical locally scaled Poisson process as a null hypothesis. The solid black line shows the plotted data. The data is presented against the distance ‘r’ between skin parasite patches measured in mm. The grey areas are simulation-envelopes around the theoretical Poisson process. Departure of the solid black line above or below the red dotted line are indicative of clustered and regular distributions instead of an independent distribution, respectively. Departure of the solid black line from the grey shaded simulation-envelops indicates significant departure from the theoretical Poisson process.

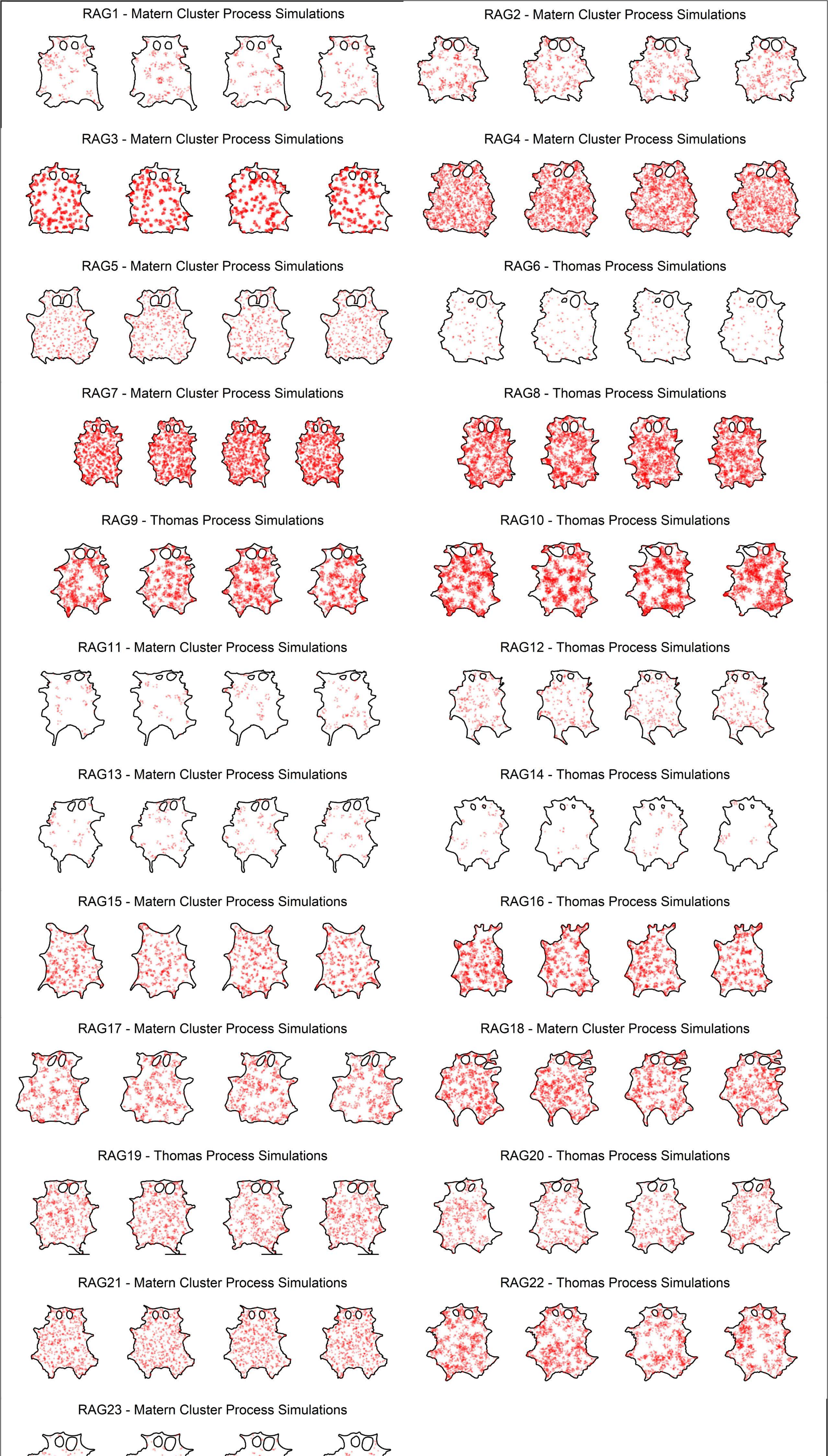

Figure S20 | Simulation output according to best fit model
Graphical output of four iterations of the respective best-fit model simulation per RAG mouse.

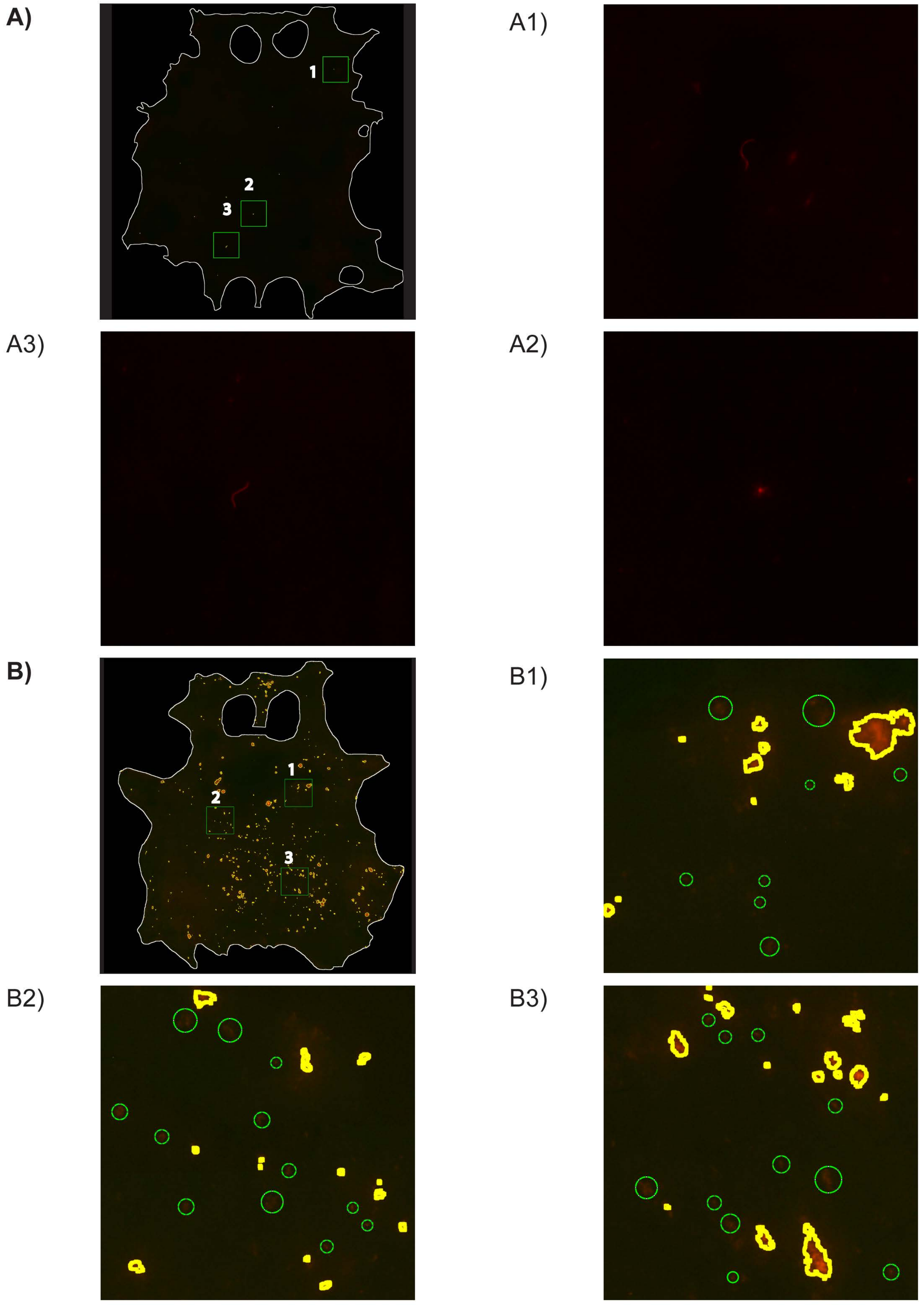

Figure S22 | False positive and negative skin parasite patch detection analysis

A) Naïve RAG mouse skin images were acquired and analyzed in the same fashion as positive skins to account for false positive detection. At close examination, detected event were identified as very small autofluorescent dust particles and other. Green squares denote the areas of enlargement in the naïve skin.

B) Manuel skin parasite patch detection in 1 cm2 areas in positive skin (marked by green squares) showed significant underestimation of small parasite patches (<0.1 mm2). Yellow lines denote parasite detected by the ImageJ macro and green dotted circles indicated areas that were manually selected as potentially being true tdTomato fluorescence and, therefore, undetected skin parasite patches.

Table S1

Statistical information on the skin parasite patch analysis

Patch Areas

Mouse IDs	Min. (μm^2)	1st Quarter (μm^2)	Median (μm^2)	Mean (μm^2)	3rd Quarter (μm^2)	Max. (μm^2)	Linear, P-Value	Outcome	Log, P-Value	Outcome	Skewness	Outcome	S.E. of Skewness	Test Statistic	Outcome	Total Patch Area (cm^2)	Total Skin Area (cm^2)	Patch Ratio of Skin (%)
**RAG1**	4150.4	6456.1	12912.2	133496.4	53032.4	6942633.5	<0.0001	Non-Normal	<0.0001	Non-Normal	10.13	Skewed	0.159	63.799	Skewed	0.314	78.212	0.401
**RAG2**	4150.4	5995.0	11528.8	34514.7	27207.9	2634557.6	<0.0001	Non-Normal	<0.0001	Non-Normal	14.83	Skewed	0.111	133.469	Skewed	0.167	57.729	0.289
**RAG3**	4150.4	5072.7	6917.3	3027315.9	13373.4	5310213076.6	<0.0001	Non-Normal	<0.0001	Non-Normal	42.25	Skewed	0.058	730.571	Skewed	54.219	66.828	81.132
**RAG4**	4189.1	7214.6	15942.0	534472.1	61178.8	286399016.2	<0.0001	Non-Normal	<0.0001	Non-Normal	28.16	Skewed	0.053	533.432	Skewed	11.491	69.068	16.637
**RAG5**	4189.1	9454.6	21003.9	111904.8	84190.0	2603663.4	<0.0001	Non-Normal	<0.0001	Non-Normal	5.51	Skewed	0.109	50.750	Skewed	0.566	73.492	0.770
**RAG6**	4189.1	6749.2	13032.9	38610.9	31069.4	586478.8	<0.0001	Non-Normal	<0.0001	Non-Normal	4.81	Skewed	0.212	22.730	Skewed	0.051	77.209	0.066
**RAG7**	4189.1	6632.8	13382.0	290149.8	47476.9	173631707.6	<0.0001	Non-Normal	<0.0001	Non-Normal	37.49	Skewed	0.053	711.487	Skewed	6.261	70.762	8.849
**RAG8**	4189.1	6981.9	13498.3	383602.7	42473.2	314545112.0	<0.0001	Non-Normal	<0.0001	Non-Normal	35.30	Skewed	0.051	698.310	Skewed	8.995	71.296	12.617
**RAG9**	4189.1	6167.3	12683.8	254524.9	44335.0	86460824.7	<0.0001	Non-Normal	<0.0001	Non-Normal	24.02	Skewed	0.064	374.435	Skewed	3.703	80.021	4.628
**RAG10**	4189.1	5469.1	8611.0	1882972.3	20480.2	4361937113.3	<0.0001	Non-Normal	<0.0001	Non-Normal	49.50	Skewed	0.049	1002.298	Skewed	46.265	82.409	56.140
**RAG11**	4305.5	8320.1	20596.6	214709.3	72029.8	10471439.6	<0.0001	Non-Normal	<0.0001	Non-Normal	8.87	Skewed	0.233	38.148	Skewed	0.232	62.160	0.373
**RAG12**	4189.1	8000.1	18967.5	62696.1	49455.1	5809980.3	<0.0001	Non-Normal	<0.0001	Non-Normal	15.81	Skewed	0.146	108.579	Skewed	0.176	57.105	0.307
**RAG13**	4189.1	9949.2	30720.3	159295.1	84655.4	3995619.6	<0.0001	Non-Normal	<0.0001	Non-Normal	5.65	Skewed	0.206	27.485	Skewed	0.221	58.000	0.382
**RAG14**	4189.1	9541.9	21527.5	49925.1	49193.2	705287.3	<0.0001	Non-Normal	0.0266	Non-Normal	5.25	Skewed	0.276	19.047	Skewed	0.038	64.535	0.059
**RAG15**	4189.1	6138.2	11229.2	433597.0	58589.7	70715614.0	<0.0001	Non-Normal	<0.0001	Non-Normal	14.55	Skewed	0.086	168.742	Skewed	3.486	40.401	8.629
**RAG16**	4189.1	6167.3	11403.8	256916.4	43520.4	23751228.3	<0.0001	Non-Normal	<0.0001	Non-Normal	10.22	Skewed	0.072	141.489	Skewed	2.947	51.233	5.752
**RAG17**	4189.1	6400.1	12800.1	232102.4	64524.3	20451586.8	<0.0001	Non-Normal	<0.0001	Non-Normal	10.17	Skewed	0.082	123.584	Skewed	2.049	47.587	4.307
**RAG18**	4189.1	6749.2	14312.9	252552.2	55506.0	53973854.8	<0.0001	Non-Normal	<0.0001	Non-Normal	19.34	Skewed	0.068	285.988	Skewed	3.306	56.286	5.873
**RAG19**	4189.1	6981.9	20014.8	741849.7	179027.3	91466142.1	<0.0001	Non-Normal	<0.0001	Non-Normal	14.92	Skewed	0.090	166.476	Skewed	5.519	64.264	8.589
**RAG20**	4189.1	8843.7	33280.3	481798.2	210678.5	29861546.3	<0.0001	Non-Normal	<0.0001	Non-Normal	10.27	Skewed	0.105	97.610	Skewed	2.597	56.540	4.593
**RAG21**	4189.1	8611.0	28218.5	658429.5	128030.4	113190876.7	<0.0001	Non-Normal	<0.0001	Non-Normal	17.22	Skewed	0.088	196.464	Skewed	5.123	59.912	8.550
**RAG22**	4150.4	6917.3	17984.9	1410841.9	75167.7	1053270594.4	<0.0001	Non-Normal	<0.0001	Non-Normal	31.89	Skewed	0.069	459.555	Skewed	17.537	73.533	23.849
**RAG23**	4150.4	8300.7	21674.1	111772.8	103759.1	1681819.0	<0.0001	Non-Normal	<0.0001	Non-Normal	4.34	Skewed	0.203	21.408	Skewed	0.160	72.675	0.220

Skin Parasites

Mouse IDs	Min.	1st Quarter	Median	Mean	3rd Quarter	Max.	Linear, P-Value	Outcome	Log, P-Value	Outcome	per cm^2	per Skin (cm^2)
**RAG1**	251.0	2290.0	6648.1	10570.2	12629.7	43123.5	0.0001	Non-Normal	0.6208	Normal	23513.0	1839000
**RAG2**	1358.6	3503.0	5528.4	9424.0	9158.3	79325.4	<0.0001	Non-Normal	0.1697	Normal	19552.7	1128754
**RAG3**	193055.3	3662059.8	5266551.5	8605329.5	9368950.5	34302760.0	0.0002	Non-Normal	0.0945	Normal	18626615.7	1244782320
**RAG4**	6164.2	23319.2	39409.3	67356.2	99910.3	335843.0	<0.0001	Non-Normal	0.8846	Normal	139381.9	9626886
**RAG5**	630.3	2643.5	4830.5	21383.9	11606.3	287609.7	<0.0001	Non-Normal	0.1611	Normal	17084.4	1255562
**RAG6**	2780.3	5588.8	9640.3	16032.1	15559.0	82828.4	<0.0001	Non-Normal	0.4119	Normal	34095.8	2632497
**RAG7**	6126.3	49168.5	78845.5	119731.5	163596.8	353988.8	0.0014	Non-Normal	0.1613	Normal	278859.1	19732605
**RAG8**	3933.0	41787.6	77590.1	93149.6	120658.0	247062.3	0.2359	Normal	0.0256	Non-Normal	274418.8	19564890
**RAG9**	473.9	11507.2	26347.2	45588.5	48604.2	181003.6	0.0012	Non-Normal	0.3487	Normal	93184.3	7456728
**RAG10**	17819.6	189430.1	469938.6	745577.4	846442.4	2548114.0	0.0019	Non-Normal	0.6373	Normal	1662067.6	136969139
**RAG11**	982.4	3056.5	5428.4	12199.7	11178.5	69469.2	<0.0001	Non-Normal	0.8334	Normal	19199.2	1193415
**RAG12**	1309.1	3249.3	5024.9	5847.2	7925.7	15235.3	0.0943	Normal	0.9993	Normal	17771.9	1014865
**RAG13**	2056.1	4884.9	11918.5	13116.8	20004.5	34186.7	0.1923	Normal	0.1010	Normal	42152.9	2444884
**RAG14**	1433.8	3116.7	4589.6	5488.3	7350.7	13219.8	0.0299	Non-Normal	0.8777	Normal	16232.6	1047571
**RAG15**	4131.5	8386.6	17612.0	19527.3	30196.7	35784.0	0.0367	Non-Normal	0.0676	Normal	62289.9	2516581
**RAG16**	7056.4	15003.7	37690.8	38286.2	52103.9	96301.9	0.1778	Normal	0.3773	Normal	133304.1	6829598
**RAG17**	3428.2	5491.5	9724.7	12697.1	14388.8	44514.5	0.0014	Non-Normal	0.7918	Normal	34393.9	1636704
**RAG18**	0.0	5010.2	22375.0	26836.9	41955.1	70036.3	0.0752	Normal	0.0001	Non-Normal	79135.2	4454194
**RAG19**	66263.1	182743.0	311323.8	325422.6	378153.2	802803.1	0.0574	Normal	0.6473	Normal	1101082.7	70760337
**RAG20**	35844.2	144539.6	232849.0	305971.0	337057.3	1375823.9	0.0001	Non-Normal	0.8655	Normal	823534.7	46562927
**RAG21**	49846.0	158783.8	353309.4	747667.7	524852.8	5646642.5	<0.0001	Non-Normal	0.4689	Normal	1249576.4	74865201
**RAG22**	12411.1	319686.2	432618.4	1011280.0	718135.4	5146964.0	<0.0001	Non-Normal	0.0947	Normal	1530074.8	112511713
**RAG23**	23671.2	64577.8	84107.0	86370.6	93306.0	232578.5	0.0126	Non-Normal	0.3278	Normal	297467.8	21618395

Patch Densities / Patch Counts

Mouse IDs	Skewness	Outcome	S.E. of Skewness	Test Statistic	Outcome	Patch Counts per cm^2	Patch Counts per skin
**RAG1**	2.35	Skewed	0.159	14.800	Skewed	3.0	235
**RAG2**	1.25	Skewed	0.111	11.250	Skewed	8.4	483
**RAG3**	8.65	Skewed	0.058	149.572	Skewed	26.8	1791
**RAG4**	2.16	Skewed	0.053	40.917	Skewed	31.1	2150
**RAG5**	1.64	Skewed	0.109	15.105	Skewed	6.9	506
**RAG6**	3.42	Skewed	0.212	16.161	Skewed	1.7	131
**RAG7**	2.34	Skewed	0.053	44.409	Skewed	30.5	2158
**RAG8**	2.73	Skewed	0.051	54.005	Skewed	32.9	2345
**RAG9**	3.82	Skewed	0.064	59.548	Skewed	18.2	1455
**RAG10**	6.07	Skewed	0.049	122.908	Skewed	29.8	2457
**RAG11**	1.87	Skewed	0.233	8.042	Skewed	1.7	108
**RAG12**	2.83	Skewed	0.146	19.436	Skewed	4.9	280
**RAG13**	1.79	Skewed	0.206	8.708	Skewed	2.4	139
**RAG14**	2.11	Skewed	0.276	7.655	Skewed	1.2	76
**RAG15**	2.62	Skewed	0.086	30.385	Skewed	19.9	804
**RAG16**	2.77	Skewed	0.072	38.349	Skewed	22.4	1147
**RAG17**	2.41	Skewed	0.082	29.286	Skewed	18.6	883
**RAG18**	2.64	Skewed	0.068	39.039	Skewed	23.3	1309
**RAG19**	2.21	Skewed	0.090	24.659	Skewed	11.6	744
**RAG20**	2.55	Skewed	0.105	24.236	Skewed	9.5	539
**RAG21**	2.20	Skewed	0.088	25.100	Skewed	13.0	778
**RAG22**	3.45	Skewed	0.069	49.717	Skewed	16.9	1243
**RAG23**	3.82	Skewed	0.203	18.843	Skewed	2.0	143

Table S2

Collective information on the point pattern analysis of skin parasite patch patterns

		Point Pattern Intensity				Quadrat Test of Inhomogeneity				Poisson Process Test		
**Mouse IDs**	**Double Point Check**	**Intensity w/o Mark (/ cm^2)**	**S.E. w/o Mark**	**Intensity by Area (/ cm^2)**	**S.E. by Area**	**Chi-Square P-values**	**Fisher's Exact P-values**	**Accepted Test**	**Outcome**	**P-values**	**Acceted Test**	**Outcome**
**RAG1**	No Doubles	3.014	1.168	0.404	0.324	0.002	0.001	Fisher's Exact	Inhomogeneous	<0.0001	Quadrat Count	Not Poisson
**RAG2**	No Doubles	8.265	2.655	0.270	0.412	0.002	0.001	Fisher's Exact	Inhomogeneous	<0.0001	Quadrat Count	Not Poisson
**RAG3**	No Doubles	26.173	9.549	80.397	3.197	0.002	0.001	Fisher's Exact	Inhomogeneous	<0.0001	Quadrat Count	Not Poisson
**RAG4**	No Doubles	31.007	10.572	16.624	4.526	0.002	0.001	Fisher's Exact	Inhomogeneous	<0.0001	Quadrat Count	Not Poisson
**RAG5**	No Doubles	6.761	2.821	0.776	0.806	0.002	0.001	Fisher's Exact	Inhomogeneous	<0.0001	Quadrat Count	Not Poisson
**RAG6**	No Doubles	1.628	0.636	0.064	0.114	0.004	0.001	Fisher's Exact	Inhomogeneous	<0.0001	Quadrat Count	Not Poisson
**RAG7**	No Doubles	30.374	10.016	8.892	3.605	0.002	0.001	Fisher's Exact	Inhomogeneous	<0.0001	Quadrat Count	Not Poisson
**RAG8**	No Doubles	32.743	13.619	12.595	4.261	0.002	0.001	Fisher's Exact	Inhomogeneous	<0.0001	Quadrat Count	Not Poisson
**RAG9**	No Doubles	18.081	5.212	4.587	1.735	0.002	0.001	Fisher's Exact	Inhomogeneous	<0.0001	Quadrat Count	Not Poisson
**RAG10**	No Doubles	29.583	14.797	56.147	4.086	0.002	0.001	Fisher's Exact	Inhomogeneous	<0.0001	Quadrat Count	Not Poisson
**RAG11**	No Doubles	1.734	0.661	0.329	0.204	0.002	0.001	Fisher's Exact	Inhomogeneous	<0.0001	Quadrat Count	Not Poisson
**RAG12**	No Doubles	4.896	1.794	0.262	0.359	0.008	0.001	Fisher's Exact	Inhomogeneous	<0.0001	Quadrat Count	Not Poisson
**RAG13**	No Doubles	2.414	0.995	0.396	0.315	0.002	0.001	Fisher's Exact	Inhomogeneous	<0.0001	Quadrat Count	Not Poisson
**RAG14**	No Doubles	1.188	0.450	0.059	0.089	0.002	0.001	Fisher's Exact	Inhomogeneous	<0.0001	Quadrat Count	Not Poisson
**RAG15**	No Doubles	19.904	8.774	8.931	3.396	0.002	0.001	Fisher's Exact	Inhomogeneous	<0.0001	Quadrat Count	Not Poisson
**RAG16**	No Doubles	22.377	8.678	5.820	3.097	0.002	0.001	Fisher's Exact	Inhomogeneous	<0.0001	Quadrat Count	Not Poisson
**RAG17**	No Doubles	18.576	7.716	4.286	2.707	0.002	0.001	Fisher's Exact	Inhomogeneous	<0.0001	Quadrat Count	Not Poisson
**RAG18**	No Doubles	23.285	9.008	5.951	3.114	0.002	0.001	Fisher's Exact	Inhomogeneous	<0.0001	Quadrat Count	Not Poisson
**RAG19**	No Doubles	11.549	4.256	8.654	2.746	0.002	0.001	Fisher's Exact	Inhomogeneous	<0.0001	Quadrat Count	Not Poisson
**RAG20**	No Doubles	9.473	4.018	4.627	2.132	0.002	0.001	Fisher's Exact	Inhomogeneous	<0.0001	Quadrat Count	Not Poisson
**RAG21**	No Doubles	13.021	4.663	8.812	2.727	0.002	0.001	Fisher's Exact	Inhomogeneous	<0.0001	Quadrat Count	Not Poisson
**RAG22**	No Doubles	16.866	7.165	23.822	3.450	0.002	0.001	Fisher's Exact	Inhomogeneous	<0.0001	Quadrat Count	Not Poisson
**RAG23**	No Doubles	1.948	0.880	0.215	0.246	0.002	0.001	Fisher's Exact	Inhomogeneous	<0.0001	Quadrat Count	Not Poisson

Table S3

Model parameter ranges

	kappa		scale	
**Models**	**Min kappa**	**Max kappa**	**Min scale**	**Max scale**
**Log-Gaussian Cox Process**	0.0005264	0.0790058	0.3797	2.7016
**Cauchy Cluster Process**	0.7338	4.8284	1.160	18.991
**Matern Cluster Process**	0.002702	0.105462	1.126	5.768
**Thomas Cluster Process**	0.002256	0.128535	0.5816	2.6680
**Variance Gamma Cluster Process**	0.0006041	0.0955190	0.649	4.063
