# Supplementary figures and images for: Spatial Point Pattern Analysis Identifies Mechanisms Shaping the Skin Parasite Landscape in Leishmania donovani Infection
Source: Front Immunol. 2021 Dec 16;12:795554. doi: 10.3389/fimmu.2021.795554 (PMC8716623; doi:10.3389/fimmu.2021.795554)

## Slide 1
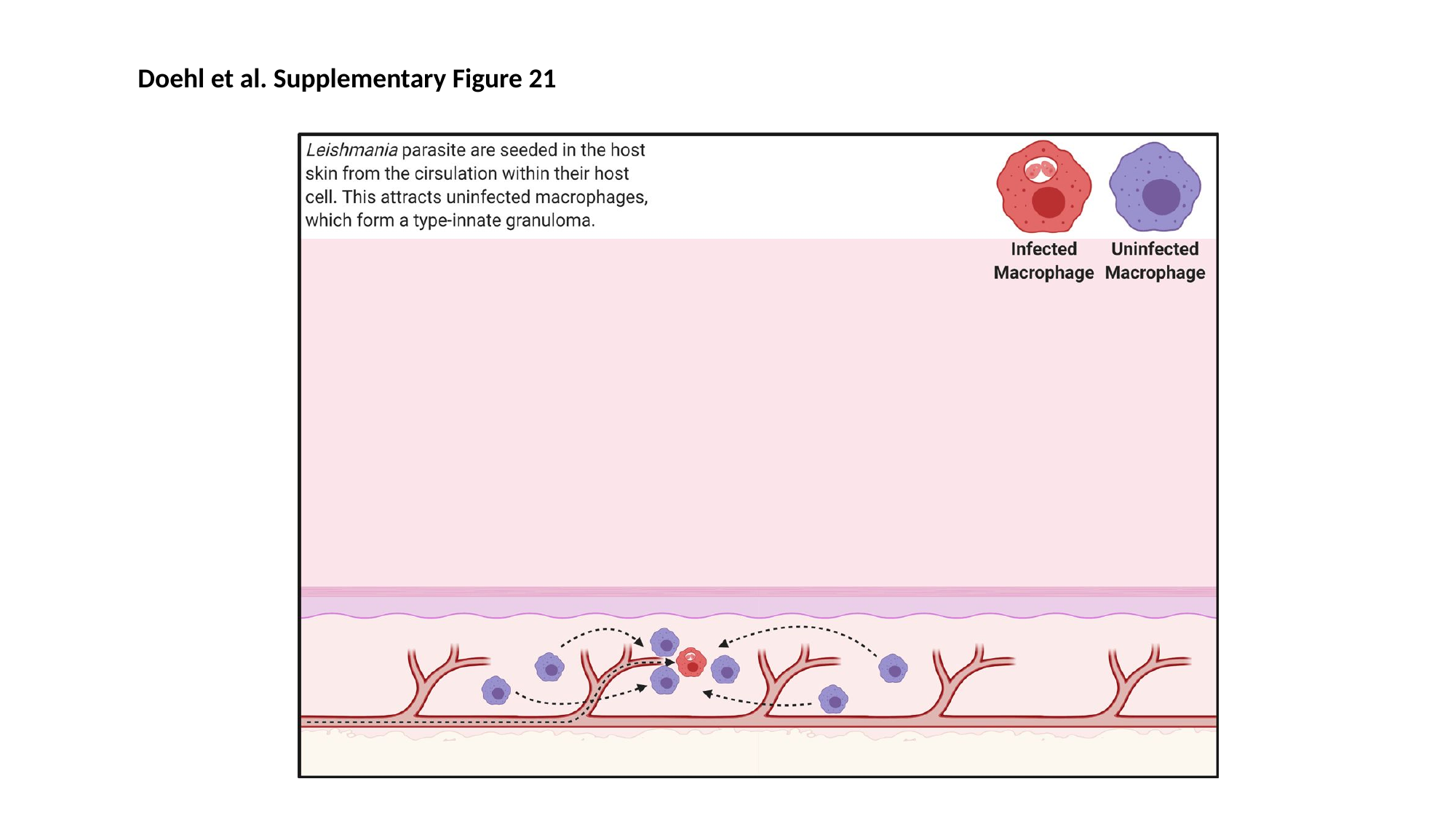

Doehl et al. Supplementary Figure 21

Supplement: Supplementary file 2 [file Presentation_1.pptx]
